# Polish adaptation of the Dimensional Anhedonia Rating Scale (DARS) - validation in the clinical sample

**DOI:** 10.3389/fpsyt.2023.1268290

**Published:** 2023-09-25

**Authors:** Aleksandra Gorostowicz, Sakina J. Rizvi, Sidney H. Kennedy, Adrian Andrzej Chrobak, Dominika Dudek, Katarzyna Cyranka, Joanna Piekarska, Eve Krawczyk, Marcin Siwek

**Affiliations:** ^1^Department of Adult Psychiatry, Jagiellonian University Medical College, Kraków, Poland; ^2^ASR Suicide and Depression Studies Unit, Department of Psychiatry, St. Michael’s Hospital, University of Toronto, Toronto, ON, Canada; ^3^Department of Psychiatry, The Ludwik Rydygier Specialist Hospital in Cracow, Kraków, Poland; ^4^Department of Adult, Child and Adolescent Psychiatry, University Hospital in Cracow, Kraków, Poland; ^5^Department of Affective Disorders, Jagiellonian University Medical College, Kraków, Poland

**Keywords:** anhedonia, validation, reliability, bipolar disorder, major depressive disorder, depression, reward, mood disorders

## Abstract

**Background:**

Anhedonia is the core symptom of depression. Its presence has been linked to worsened prognosis. The Dimensional Anhedonia Rating Scale (DARS) is a scale measuring desire, motivation, effort and consummatory pleasure across different domains. The aim of this paper was to confirm factor structure, assess reliability and validity of the Polish adaptation of the DARS in a clinical sample of patients with mood disorders and healthy controls (HC).

**Methods:**

The study sample included 161 participants aged 18–65 years - 34 HC, 72 patients with bipolar disorder and 55 with major depressive disorder (in depressive episode or remission). Reliability of the Polish adaptation of the DARS was assessed using Cronbach’s α and the average inter-item correlation (AIC). Convergent and divergent validity was established by Pearson’s correlations between the DARS and the Snaith-Hamilton Pleasure Scale (SHAPS), the Quick Inventory of Depressive Symptomatology- self-report (QIDS-SR), the Hospital Anxiety and Depression Scale (HADS). The structure of the scale was examined by factor analysis.

**Results:**

The factor structure was consistent with the original scale. Strong internal consistency for the DARS total score (Cronbach’s α = 0.95) and all subscales (0.86–0.93) was observed. The DARS demonstrated good convergent (moderate to strong correlations with measures of anhedonia and depression) and divergent validity (weak correlations with anxiety level).

**Conclusion:**

The Polish DARS demonstrated excellent internal consistency and very good validity. The scale is a valuable contribution to the psychometrics of anhedonia measures in patients with mood disorders.

## 1. Introduction

Anhedonia is defined by the DSM-5 (Diagnostic and Statistical Manual of Mental Disorders 5th edition) diagnostic criteria as “markedly diminished interest or pleasure in all, or almost all, activities of the day, nearly every day (as indicated by either subjective account or observation)” (1). Based on the currently used diagnostic classifications for major depressive disorder (MDD) anhedonia is one of the two (1, 2) or three (3) main symptoms necessary for the diagnosis of a depressive episode. Anhedonia is also present in other psychiatric and neurological conditions: schizophrenia (4), bipolar disorder (BD) (5), substance use disorders (SUDs) (6), personality disorders (7), chronic pain (8) and Parkinson’s disease (9, 10). Clinically relevant anhedonia has been observed in about 70% of patients with MDD (11). The results of one of a recent meta-analysis indicated that patients with current MDD reported significantly higher than all other groups analyzed (i.e., patients with schizophrenia, Parkinson’s disease, chronic pain, SUD and healthy controls) (10).

The presence of anhedonia has been linked to a worsened prognosis in MDD patients – longer time to remission and recovery (12, 13), poorer treatment outcomes (14, 15) and higher suicide risk (independently of other depressive symptoms) (16, 17). Accumulating evidence suggests that commonly used first-line antidepressants (i.e., SSRIs - *selective serotonin reuptake inhibitors*) do not adequately treat anhedonia (18, 19) or can even induce emotional blunting and apathy (with prevalence of SSRI-induced apathy ranging between 20 and 92%) (20).

Anhedonia is a multifaceted construct reflecting deficits in reward processing (18). Results from neurobiological studies have shown that the hedonic process consists of different components, namely: interest/desire, reward anticipation, motivation/effort and consummatory pleasure (21). Thus, anhedonia is a broad term used to describe deficits across various areas: reward liking (*consummatory anhedonia*), wanting (*motivational anhedonia*) and reward learning (learning the associations between a stimuli and an outcome, based on past rewards and punishments) (22, 23). It has been observed that different parts of reward processing are characterized by both distinct and common brain circuits and neurotransmitters – for example dopamine has been typically linked to motivational deficits, while endogenous opioids may be more important for experiencing pleasure (21, 22, 24). Many areas of reward circuitry appear to play a role in the development of anhedonia, including both subcortical (nucleus accumbens, amygdala, hippocampus, insula, lateral habenula, ventral pallidum, ventral tegmental area), and cortical structures (orbitofrontal cortex, ventromedial prefrontal cortex, anterior cingulate cortex) (11, 25).

There are 4 validated measures of anhedonia that are typically used in clinical studies: the Snaith-Hamilton Pleasure Scale (SHAPS) (26), the Fawcett-Clark Pleasure Scale (FCPS) (27), the Revised Chapman Physical Anhedonia Scale (CPAS) and the Chapman Social Anhedonia Scale (CSAS) (28). These measures differ with respect to the aspects of anhedonia they measure (e.g., consummatory pleasure, motivation, effort). Some items included in the FCPS, CSAS and CPAS are culturally biased, while the SHAPS provides responders with more general categories of hedonic events/activities (potentially not detecting the most pleasant ones) (21).

In order to overcome the limitations of the above mentioned tools, a few second-generation scales measuring anhedonia have been developed (21). One such tool is the Dimensional Anhedonia Rating Scale (DARS), a 17-item scale that assesses desire, motivation, effort and consummatory pleasure across different domains (hobbies, foods/drinks, social activities and sensory experiences) (29). The DARS is unique in that individuals provide their own examples of pleasurable activities/experiences within each domain. The DARS has been validated in both clinical and non-clinical populations, and demonstrates high reliability, good convergent and divergent validity (29–31). The DARS has also demonstrated additional utility over SHAPS in predicting treatment-resistance in a population of depressed patients (29). It has been validated in German, Spanish and Chinese (30–32).

Currently only the SHAPS and CPAS/CSAS have been translated into Polish for use in clinical or research practice (33, 34). These studies have demonstrated excellent and acceptable reliability of the SHAPS and CPAS/CSAS, respectively. To our knowledge, the DARS was validated in Polish among anonymous participants from a community sample only in the context of a 2017 Master’s thesis that is yet to be published (35). We decided to perform a separate adaptation and validation in order to: (1) conduct a translation that includes a back-translation from Polish to English by a native speaker, which is an essential step for scale translation; (2) validate the DARS in a clinical sample with mood disorders as well as healthy controls; and (3) establish convergent validity with a gold standard scale (e.g., the SHAPS). Additional goals of this study were to confirm the reliability and factor structure of the DARS in Polish.

## 2. Materials and methods

### 2.1. Participants

We enrolled patients from the Department of Adult, Child, and Adolescent Psychiatry, University Hospital in Cracow (both in- and outpatients) if they met the following inclusion criteria: age 18–65 years; met criteria for a DSM-5 diagnosis of MDD (first episode or recurrent depression, in depressive episode or remission) or bipolar disorder (BD; in depressive episode or remission); no severe or unstable medical illness; and no substance use disorder (apart from nicotine or caffeine) in the last 12 months. As psychiatric comorbidities are highly prevalent in patients with mood disorders (36, 37), we decided to enroll patients with additional diagnoses (anxiety disorders, eating disorders, personality disorders, attention deficit hyperactivity disorder [ADHD]) if their intensity was mild (i.e., not impairing a patient’s functioning distinctly and not being the primary reason for seeking medical help at the time of enrollment). Healthy controls (HC) were recruited from local volunteers. Inclusion criteria for the control group were: age 18–65; no history of psychiatric treatment; no use of psychotropic medications; no significant medical illness; no first degree family members with psychiatric diagnoses; no substance use disorder (apart from nicotine or caffeine).

The study was approved by the Bioethics Committee of the Jagiellonian University in Krakow (approval No. 1072.6120.45.2019). Patients and healthy controls were recruited between December 2019 and August 2022. All participants provided informed, written consent to take part in the study.

### 2.2. Measures

The original DARS scale was translated from English to Polish by two independent clinicians with proficiency in English (one being a native speaker of both English and Polish and another with a Master of English Philology). The final Polish version was back-translated into English by a native speaker of Polish and sent to the authors of the scale for feedback. All corrections were addressed, and the final version was accepted by the authors. The Polish version of the DARS is shown in Appendix 1.

The DARS is a self-administered 17-item scale divided into four categories – hobbies, foods/drinks, social activities (four items each) and sensory experiences (five items). For each question respondents are asked to give 2–3 examples of their own favorite activities/experiences and rate their interest, desire, motivation and pleasure “right now” on a 5-point Likert scale (Not at all = 0; Slightly = 1; Moderately = 2; Mostly = 3; Very much = 4). The final score is a sum of all items (minimum score is 0 and maximum is 68), with lower scores indicating more severe anhedonia (29).

The SHAPS is a self-administered tool with 14 items measuring pleasure from different experiences. Each question has four answers: strongly agree, agree, disagree and strongly disagree. The first two responses are rated 0 points and the last two 1 point. Total score ranges from 0 to 14 with higher scores indicating higher level of anhedonia (26).

The QIDS-SR (Quick Inventory of Depressive Symptomatology- self-report) is a 16-item scale measuring severity of depressive symptoms (during “the last 7 days”) and based on the DSM criteria for MDD. The total score ranges from 0 to 27 and higher scores indicate more severe depressive symptoms (38).

The HADS (Hospital Anxiety and Depression Scale) is a self-rated, 14-item tool measuring presence of anxiety (HADS-A: Anxiety subscale – seven items) and depression (HADS-D: Depression subscale – seven items) during the last week. Each item is scored from 0 to 3. The total scores range from 0 to 21 for each subscale, with higher scores indicating higher anxiety or depression (39, 40).

For the analysis of convergent and divergent validity it is essential to include scales assessing partially similar (i.e., depression) and different constructs (anxiety). Therefore, we decided to include additional tools measuring depressive (QIDS-SR, HAD-D) and anxiety symptoms (HAD-A).

### 2.3. Procedures

Participants enrolled in the study were assessed during one visit to the Department of Psychiatry. The medical interview was performed by a trained clinician in order to establish and verify psychiatric diagnoses (according to the DSM-5 criteria). Both patients and HC were interviewed with a structured psychiatric interview (MINI - Mini International Neuropsychiatric Interview (41)) to ensure that the inclusion criteria for each group were met. Both medical and sociodemographic data were collected. Participants were then asked to fill in the following scales using the Polish versions: DARS, SHAPS, QIDS-SR, and HADS.

### 2.4. Statistical analysis

Basic socio-demographic and clinical data are presented as mean and standard deviation (SD; for normally distributed quantitative data), median with interquartile range (IQR; for non-normally distributed quantitative data) or percentages for nominal data. Normality was assessed by the analysis of histograms and z-scores for skewness and kurtosis – values <1.96 indicate approximation of the normal distribution.

Comparisons of quantitative data between two groups were performed with an independent samples t-test or Mann–Whitney test. Differences across three groups were assessed with a one-way ANOVA or Kruskal-Wallis test, depending on the normality of the data. Bonferroni correction was applied to all post-hoc tests (pairwise comparisons).

Internal reliability of the DARS total scale and the four subscales was assessed in the group of depressed MDD and BD patients using Cronbach’s α (with values ≥0.7 considered acceptable) and the average inter-item correlation (AIC) (42, 43). Convergent and divergent validity were established by calculating correlations between the total DARS score and subscale scores and the SHAPS, QIDS-SR, HADS-A (anxiety subscale) and HADS-D (depression subscale). Pearson’s or Spearman’s rank correlation coefficient was selected based on the assessment of normality of distribution.

The structure of the ‘Polish DARS’ was examined by factor analysis using direct oblimin rotation with delta = 0 (oblique rotation method was selected due to the suspected correlation between factors). Correlation matrix (demonstrating correlations between each pair of items) was checked for values lower than 0.3 or higher than 0.9. The determinant was also examined to avoid multicollinearity (value >0.00001 would be accepted). Sampling adequacy was assessed with a Kaiser-Meyer-Olkin (KMO) measure and Bartlett’s test (42). The number of factors for extraction was determined using Kaiser’s criteria (44) and analysis of a scree plot (Cattell’s method) (45). As each of these two methods yields different results, a confirmatory factor analysis (CFA) was performed to compare models. The following model fit indices were selected to compare models: root mean square error of approximation (RMSEA), comparative fit index (CFI), Tucker Lewis index (TLI), standardized root mean square residual (SRMR). Values for CFI and TLI higher than 0.95, for RMSEA <1.0 and for SRMR <0.08 were considered accurate (indicating good fit of the model) (46–48).

Statistical analyses were carried out using Statistical Package for the Social Sciences (SPSS) version 28.0. CFA was performed with IBM AMOS version 28. The level of significance was set at *p* < 0.05.

## 3. Results

### 3.1. Sample characteristics

In total 161 Caucasian participants were included in the study – 72 patients with BD, 55 patients with MDD and 34 HC. A total of 70.8% of the BD group and 89.1% of the MDD group met criteria for a current depressive episode. The remaining patients were in clinical remission. Among the BD patients, 15 had type I, 50 had type II and 7 others had unspecified BD. Basic socio-demographic and clinical data are presented in Table 1. Distributions of age, gender, level of education, presence of medical disorders and psychiatric comorbidities were not statistically different across the BD and MDD groups. There was a significant difference between BD and MDD patients in median duration of illness (longer duration for BD patients – 7 vs. 4.5 years, *p* = 0.025).

**Table 1:** The distribution of basic socio-demographic and clinical data across groups of BD, MDD patients and healthy controls.

	Bipolar Disorder (*n* = 72)	Major Depressive Disorder (*n* = 55)	Healthy controls (*n* = 34)	*p*
Age (years: median; IQR)	37 (19)	30 (14)	30.5 (15.5)	0.146^a^
Gender (n female; %female)	46 (63.9%)	34 (61.8%)	18 (52.9%)	0.551^b^
Education level (%higher degree completed)	66.7%	70.5%	80.8%	0.709^c^
Medical illnesses (%yes)	29.2%	30.9%	20.6%	0.564^b^
- Hypothyroidism	15.3%	10.9%	2.9%	
- Hypertension	6.9%	7.3%	2.9%	
- Diabetes type 2	5.6%	7.3%	0	
- Cardiac arrythmias	2.8%	1.8%	2.9%	
- Hypercholesterolemia	2.8%	3.6%	0	
- Irritable Bowel Syndrome	2.8%	1.8%	0	
- Asthma	2.8%	3.6%	0	
- Epilepsy	1.4%	0	0	
- Peptic Ulcer Disease	1.4%	0	0	
- Polycystic Ovarian Syndrome	0	1.8%	0	
- Ulcerative Colitis	0	0	2.9%	
- Migraine	1.4%	0	2.9%	
- Diabetes type 1	0	0	2.9%	
- Acne	0	0	2.9%	
Duration of illness; (years: median; IQR)	7 (9)	4.5 (6.75)	n/a	**0.025** ^**d**^
Other psychiatric diagnoses (%yes)	25%	32.7%	n/a	0.104^b^
- ADHD	9.7%	7.3%		
- Anxiety disorder	5.6%	16.4%		
- Eating disorders	1.4%	3.6%		
- Personality disorders	15.3%	10.9%		

ADHD, attention deficit hyperactivity disorder; IQR, interquartile range. Bold *p*-values represent statistically significant results.

a Kruskal-Wallis test.

b Chi-square test.

c Fisher’s Exact test.

d Mann–Whitney test.

### 3.2. Distribution of anhedonia scores

The DARS scores (total and subscales) and SHAPS scores in BD and MDD patients in a current depressive episode, as well as HCs is presented in Table 2. Statistically significant differences in all measures were observed when comparing BD or MDD depressed patients with HC. No significant differences were shown when comparing anhedonia between BD and MDD depressed patients.

**Table 2:** Comparison of the distribution of anhedonia rating scales (DARS – total and subscales; SHAPS) in BD, MDD (both in a current depressive episode) and HC groups.

	Bipolar Disorder (*n* = 51)	Major Depressive Disorder (*n* = 49)	Healthy controls (*n* = 34)	*p*
DARS total score (mean; SD)	41.4 (14.6)	37.3 (16.3)	58.4 (5.9)	**<0.001** **BD vs. HC: <0.001** **MDD *vs* HC: <0.001** BD vs. MDD: 0.404
DARS – hobbies (median; IQR)	12 (7)	8.5 (6)	15.5 (3)	**<0.001** **BD vs. HC: <0.001** **MDD vs. HC: <0.001** BD vs. MDD: 0.138
DARS – Food/drink (median; IQR)	11 (5)	9 (7)	13 (3.3)	**<0.001** **BD vs. HC: <0.001** **MDD vs. HC: <0.001** BD vs. MDD: 0.586
DARS- Social (median; IQR)	10 (6)	8 (8)	14 (4)	**<0.001** **BD vs. HC: <0.001** **MDD vs. HC: <0.001** BD vs. MDD: >0.999
DARS – Sensory (median; IQR)	14 (9)	12.5 (9.5)	18 (6)	**<0.001** **BD vs. HC: <0.001** **MDD vs. HC: <0.001** BD vs. MDD: >0.999
SHAPS (median; IQR)	4 (7)	5 (8)	0 (0)	**<0.001** **BD vs. HC: <0.001** **MDD vs. HC: <0.001** BD vs. MDD: 0.895

For the DARS total score one-way independent ANOVA was applied. All other comparisons were performed with Kruskal-Wallis test. Bonferroni correction was applied to all post-hoc tests (pairwise comparisons between the subgroups). BD, bipolar disorder; HC, healthy controls; IQR, interquartile range; MDD, major depressive disorder; SD, standard deviation. Bold *p*-values represent statistically significant results.

**Figure 1:**
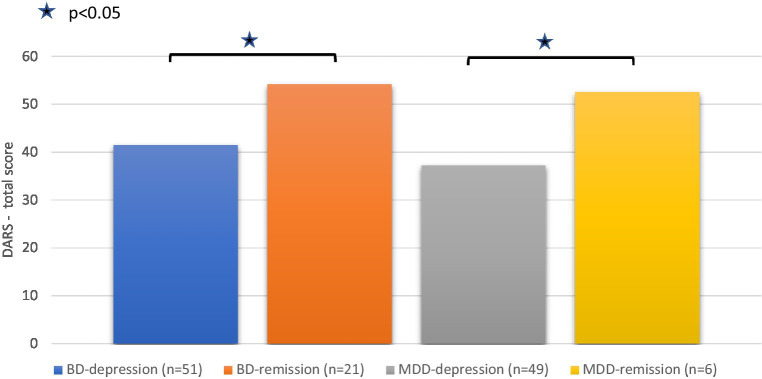
The DARS total score in the subgroups of BD and MDD patients (depressed and remitted). For comparing subgroups in BD and MDD patients The Independent samples *t*-test and Mann–Whitney test were used, respectively.

When the DARS total score was compared within subpopulations of patients in a current depressive episode with those in remission, statistically significant differences in mean DARS score were observed between BD depressed vs. BD remitted patients [41.4 vs. 54.2; *t*(67.9) = 5.18, *p* < 0.001] and between MDD depressed vs. MDD remitted patients (37.3 vs. 52.5; Mann–Whitney U = 61, *p* = 0.023; Figure 1). No statistically significant differences were shown when comparing DARS total score between remitted BD and MDD patients (U = 57, *p* = 0.76).

A median DARS total score was compared between patients with psychiatric comorbidities and without. In the BD depressed group, a statistically significant lower DARS score (indicating higher anhedonia) was observed among those with comorbidities (31 vs. 49.5, Mann–Whitney U = 359.5, *p* = 0.023). This difference was not observed in MDD depressed patients (43 vs. 32, Mann–Whitney U = 133.5, *p* = 0.248).

### 3.3. Reliability analysis

Reliability analysis was performed in the depressed BD and MDD groups. Internal consistency as measured by Cronbach’s α was high for the DARS total score (0.95) and all subscales: hobbies (0.92), foods/drinks (0.86), social activities (0.92) and sensory experiences (0.93). Analysis for Cronbach’s α after deletion of an item showed that no items would significantly increase α if deleted. The AIC for total score was 0.55 and for the subscales: 0.74 (hobbies), 0.61 (foods/drinks), 0.75 (social activities) and 0.73 (sensory experiences).

### 3.4. Validity analysis

Validity analyses were performed in the group of depressed BD and MDD patients – results are presented in Table 3. The DARS total score was strongly correlated with SHAPS (r_S_ = −0.72, *p* < 0.001) and HADS-D (r_S_ = −0.72, *p* < 0.001) scores, moderately correlated with QIDS (r_S_ = −0.55, *p* < 0.001) and weakly correlated with HADS-A (r_S_ = −0.29, *p* = 0.005; negative correlation coefficients due to the fact that lower scores on the DARS represent higher level of anhedonia). All the DARS subscales were moderately correlated with SHAPS, QIDS, HADS-D, and weakly correlated with HADS-A score.

**Table 3:** Convergent and divergent validity of the DARS total score and subscales in depressed BD and MDD patients.

	SHAPS	QIDS	HADS-A	HADS-D
DARS-total score	−0.72^**^	−0.55^**^	−0.29^**^	−0.72^**^
DARS -hobbies	−0.56^**^	−0.40^**^	−0.12	−0.56^**^
DARS-foods/drinks	−0.64^**^	−0.44^**^	−0.31^**^	−0.50^**^
DARS- social activities	−0.65^**^	−0.50^**^	−0.26^*^	−0.67^**^
DARS-sensory experiences	−0.62^**^	−0.48^**^	−0.23^*^	−0.64^**^

* Correlations significant at *p* < 0.05.

** Correlations significant at *p* < 0.01.

### 3.5. Factor analysis

All participants were included in the factor analysis [MDD, BD patients (depressed and remitted) and HCs]. To examine the internal structure of the Polish version of the DARS, factor analysis was performed with direct oblimin rotation. Analysis of the correlation matrix and the determinant indicated lack of significant multicollinearity. KMO measure was 0.936 and Bartlett’s test was statistically significant (*χ*^2^ = 2,358,5; df = 136; *p* < 0.001) which indicated sampling adequacy. Kaiser’s criteria demonstrated a three-factor structure of the scale (with the fourth factor’s eigenvalue being >0.9), but the scree plot would allow retention of four factors. Therefore, CFA was performed for the three- and four-factor model. The model fit indices for the three-factor model were: RMSA = 0.098, CFI = 0.926, TLI = 0.913, SRMR = 0.0519 and for the four-factor model: RMSA = 0.072, CFI = 0.961, TLI = 0.953, SRMR = 0.0428. The model with four factors demonstrated a better fit and was analogous to the original construct of the scale – the factors corresponded to the DARS subscales (with 79.5% of variance explained) (29). Factor loadings for each item are presented in Table 4 – all values are greater than 0.4 which is substantial (42).

**Table 4:** Individual factor loadings for all items (values reported from the Pattern matrix).

	Factor: hobbies	Factor: foods/drinks	Factor: social	Factor: sensory
Item 1	0.778			
Item 2	0.918			
Item 3	0.881			
Item 4	0.917			
Item 5		0.453		
Item 6		0.454		
Item 7		0.743		
Item 8		0.930		
Item 9			0.768	
Item 10			0.854	
Item 11			0.928	
Item 12			0.758	
Item 13				0.631
Item 14				0.855
Item 15				0.866
Item 16				0.891
Item 17				0.774

### 3.6. Self-reported examples

We collected the most common examples of activities/experiences provided by the participants for each of the subscale (Table 5).

**Table 5:** The most commonly provided examples of favorite activities/experiences.

Hobbies	Reading, running, watching television/films, playing computer games, dancing, hiking, going to the gym, cycling, walking a dog
Foods/drinks	Pizza, sushi, dumplings, coffee, tea, pasta
Social	Playing games, going to cinema, cooking with partner, going out and chatting with friends, taking a walk with family/friends, playing with kids
Sensory experiences	Petting animals (cats/dogs), listening to music, massage, smell of perfume, listening to sea waves, watching sunset

## 4. Discussion

This study demonstrated that the psychometrics of the Polish DARS were strong, and the factor structure was consistent with the original scale (29). The reliability of the Polish DARS is in line with published data on the German (31), Spanish (30), and English (29) versions of the DARS (Cronbach’s α = 0.86, 0.92, and 0.96, respectively). The AIC for the total score and subscales was also similar to the English version of the DARS (29).

The Polish DARS also demonstrated very good convergent validity – the scores of the total scale and the subscales correlated moderately to strongly with other included measures of anhedonia (SHAPS) and depression (QIDS total score, and HADS-D total score). As anhedonia is a core symptom of depression, a moderate correlation between scales measuring these two features is not surprising. However, higher than expected correlations were observed between the DARS (total and subscales) and HADS-D, with coefficients in the range from 0.50 to 0.72. This can be potentially explained by the fact that HADS-D incudes only 7 questions about depressive symptoms and nearly half of them refer to anhedonia (“I still enjoy the things I used to enjoy:”; “I look forward with enjoyment to things:”; “I can enjoy a good book or radio or TV program:”). The Polish DARS has also shown good divergent validity as it was only weakly correlated with the level of anxiety (measured by HADS-A). The validity results with the SHAPS are similar to those in other language versions of the DARS – very strong correlation with the SHAPS was observed in the original scale (r_S_ = −0.79) and strong in the Spanish (r = −0.51) and German versions (r_S_ = −0.50).

The Polish version of the DARS showed an ability to detect anhedonia as a “state”; the DARS total scores were statistically significantly lower in depressed MDD and BD patients when compared to those in remission (indicating higher anhedonia in the depressive phase of illness). The scale also demonstrated potential clinical utility in discriminating patients with mood disorders from controls; statistically significant differences in the distribution of the DARS (total scale and subscales) between BD/MDD depressed patients and HC were observed. The DARS total score differed significantly between BD depressed patients with and without psychiatric comorbidities - patients with comorbidities were observed to present higher anhedonia (lower DARS score).

The examples of pleasurable activities/sensations were similar to the ones listed in the original scale (29) with the exception of foods – in the Polish version participants named a few dishes typical of Polish cuisine: dumplings, stuffed cabbage, “Polish sour soup.” The examples provided support the ability of participants to produce examples that matched what each DARS domain measured.

We are aware of several limitations in our work: (1) only patients with mood disorders and healthy controls were included, which limits the generalizability of the findings to a wider psychiatric population. However, anhedonia is a core symptom of depression, so the aim of our study was to validate the DARS firstly in the group of BD and MDD patients. (2) The study only included self-rated scales. To minimize the impact of this limitation and not to rely solely on one tool, we used a variety of scales to assess convergent and divergent validity. (3) We included patients with psychiatric comorbidities. However, comorbidity is very common in the population of patients with mood disorders (36, 37). Thus, including a population with a diagnosis not limited to only MDD or BD makes our clinical sample more naturalistic. (4) Patients with mood disorders were on different pharmacotherapy regimens, which could have impacted their DARS scores. (5) The divergent validity was tested using only the anxiety subscale of the HADS tool. (6) The sample size is relatively small. However, the subject to item ratio obtained was around 9.5:1 which is close to the optimal recommended value of 10:1 (49).

Anhedonia is a common symptom of mood disorders with an important impact on prognosis. Therefore, it is clinically relevant to adequately measure anhedonia, taking into account its different dimensions and complex nature. The Polish version of the DARS demonstrated excellent internal consistency and very good validity, comparable to the original scale. Taking into consideration the limited number of tools to measure anhedonia available in Polish, the DARS is a valuable contribution to the psychometrics of anhedonia measures in patients with mood disorders.

## Data availability statement

The raw data supporting the conclusions of this article will be made available by the authors, without undue reservation.

## Ethics statement

The studies involving humans were approved by the Bioethics Committee of the Jagiellonian University in Krakow. The studies were conducted in accordance with the local legislation and institutional requirements. The participants provided their written informed consent to participate in this study.

## Author contributions

AG: Conceptualization, Data curation, Formal analysis, Investigation, Methodology, Project administration, Resources, Visualization, Writing – original draft, Writing – review and editing. SR: Methodology, Supervision, Writing – review and editing. SK: Methodology, Supervision, Writing – review and editing. AC: Conceptualization, Investigation, Methodology, Project administration, Resources, Writing – review and editing. DD: Conceptualization, Investigation, Methodology, Project administration, Resources, Supervision, Writing – review and editing. KC: Conceptualization, Investigation, Methodology, Writing – review and editing. JP: Conceptualization, Investigation, Methodology, Writing – review and editing. EK: Conceptualization, Investigation, Methodology, Writing – review and editing. MS: Conceptualization, Investigation, Methodology, Project administration, Resources, Supervision, Writing – review and editing.
