# Supplementary material for: Polish adaptation of the Dimensional Anhedonia Rating Scale (DARS) - validation in the clinical sample
Source: Front Psychiatry. 2023 Sep 25;14:1268290. doi: 10.3389/fpsyt.2023.1268290 (PMC10561247; doi:10.3389/fpsyt.2023.1268290)
Supplement: Supplementary file 1 [file Data_Sheet_1.docx]

Appendix 1

**DARS - Dimentional Anhedonia Rating Scale (Skala Oceny Wymiarów Anhedonii)**

**Instrukcja**: Zastanów się uważnie i podaj co najmniej 2 przykłady przyjemnych aktywności/doświadczeń dla każdej z kategorii. Nawet jeśli ostatnio nie miałeś/-aś przyjemności z aktywności/doświadczeń, użyj aktywności/doświadczeń, z których pamiętasz, że cieszyłeś/-aś się najbardziej i odpowiedz na pytania podając w jakim stopniu dotyczą one Ciebie w tej chwili. Zaznacz pole, które najlepiej opisuje Twoje samopoczucie.

1. **Proszę podaj przynajmniej 2 z Twoich ulubionych rozrywek/hobby, które**

**NIE są aktywnościami głównie towarzyskimi (np. czytanie, granie w gry komputerowe, bieganie):**

**Myśląc teraz o tych aktywnościach:**

1. Te aktywności sprawiałyby mi przyjemność.

**Zdecydowanie nie**

**Minimalnie**

**Umiarkowanie**

**Przeważnie**

**Bardzo**

1. Spędzał(a)bym czas wykonując te aktywności.

**Zdecydowanie nie**

**Minimalnie**

**Umiarkowanie**

**Przeważnie**

**Bardzo**

1. Chcę wykonywać te aktywności.

**Zdecydowanie nie**

**Minimalnie**

**Umiarkowanie**

**Przeważnie**

**Bardzo**

1. Interesowałyby mnie te aktywności.

**Zdecydowanie nie**

**Minimalnie**

**Umiarkowanie**

**Przeważnie**

**Bardzo**

1. **Proszę podaj przynajmniej 2 z Twoich ulubionych potraw lub napojów (np. pierogi, pizza, sok pomarańczowy, kawa)**:

**Myśląc teraz o tych potrawach/napojach:**

1. Podjąłbym/podjęłabym wysiłek aby zdobyć/przygotować te potrawy/napoje.

**Zdecydowanie nie**

**Minimalnie**

**Umiarkowanie**

**Przeważnie**

**Bardzo**

1. Te potrawy/napoje sprawiłyby mi przyjemność.

**Zdecydowanie nie**

**Minimalnie**

**Umiarkowanie**

**Przeważnie**

**Bardzo**

1. Chcę mieć te potrawy/napoje.

**Zdecydowanie nie**

**Minimalnie**

**Umiarkowanie**

**Przeważnie**

**Bardzo**

1. Zjadł(a)bym tyle tych potraw ile bym zdołał(a).

**Zdecydowanie nie**

**Minimalnie**

**Umiarkowanie**

**Przeważnie**

**Bardzo**

1. **Proszę podaj przynajmniej 2 z Twoich ulubionych towarzyskich aktywności (np. pójście do kina ze znajomymi, zabawa z dziećmi/wnukami, wspólne gotowanie z partnerem/partnerką/przyjaciółmi):**

**Myśląc teraz o tych aktywnościach:**

1. Spędzanie czasu na robieniu tych rzeczy uszczęśliwiłoby mnie.

**Zdecydowanie nie**

**Minimalnie**

**Umiarkowanie**

**Przeważnie**

**Bardzo**

1. Był(a)bym zainteresowany(a) robieniem rzeczy, które angażują innych ludzi.

**Zdecydowanie nie**

**Minimalnie**

**Umiarkowanie**

**Przeważnie**

**Bardzo**

1. Był(a)bym tym, który(a) planował(a)by te aktywności.

**Zdecydowanie nie**

**Minimalnie**

**Umiarkowanie**

**Przeważnie**

**Bardzo**

1. Aktywnie uczestniczył(a)bym w tych towarzyskich aktywnościach.

**Zdecydowanie nie**

**Minimalnie**

**Umiarkowanie**

**Przeważnie**

**Bardzo**

1. **Proszę podaj przynajmniej 2 z Twoich ulubionych doświadczeń zmysłowych (np. zapach bryzy morskiej, perfum; masaż, głaskanie zwierząt; słuchanie muzyki; oglądanie zachodu słońca):**

**Myśląc teraz o tych doświadczeniach:**

1. Aktywnie poszukiwał(a)bym tych doświadczeń.

**Zdecydowanie nie**

**Minimalnie**

**Umiarkowanie**

**Przeważnie**

**Bardzo**

1. Z podekscytowaniem myślę o tych doświadczeniach.

**Zdecydowanie nie**

**Minimalnie**

**Umiarkowanie**

**Przeważnie**

**Bardzo**

1. Gdybym miał(a) mieć te doświadczenia, rozkoszował(a)bym się każdą chwilą.

**Zdecydowanie nie**

**Minimalnie**

**Umiarkowanie**

**Przeważnie**

**Bardzo**

1. Chcę mieć takie doświadczenia.

**Zdecydowanie nie**

**Minimalnie**

**Umiarkowanie**

**Przeważnie**

**Bardzo**

1. Starał(a)bym się poświęcić czas na te doświadczenia.

**Zdecydowanie nie**

**Minimalnie**

**Umiarkowanie**

**Przeważnie**

**Bardzo**
